# Intranasal delivery of regenerative biologics for neurological disorders: a systematic review of clinical studies

**DOI:** 10.3389/fddev.2026.1969111

**Published:** 2026-10-16

**Authors:** Umm E Habiba, Iqra Sarwar, Sabiha Shamim, David Lawrence Greene

**Affiliations:** 1 Department of Research and Development, R3 Stem Cell LLC, Scottsdale, AZ, United States; 2 R3 Stem Cell LLC, Scottsdale, AZ, United States; 3 Department of Research and Development, R3 Medical Research LLC, Office 1B, Islamabad, Federal Territory, Pakistan

**Keywords:** blood brain barrier, central nervous system, exosomes, extracellular vesicles, neural stem cells, neurodegenerative diseases, regenerative medicine

## Abstract

**Introduction:**

Intranasal delivery is a minimally invasive route for administering regenerative biologics to the central nervous system while bypassing the blood-brain barrier. Human clinical evidence for this approach across neurological disorders remains dispersed across multiple disease areas, biologic classes, dosing frameworks, and delivery methods, limiting field-level synthesis. This study was conducted to systematically evaluate the safety, dosing characteristics, delivery method heterogeneity, and disease-specific direction of effect of intranasal regenerative biologics, including mesenchymal stromal cells (MSCs), neural stem cells (NSCs), exosomes, secretome and conditioned medium, breast milk-derived biologic preparations, and related regenerative products, in patients with neurological disorders.

**Methodology:**

An International Prospective Register of Systematic Reviews (PROSPERO)-registered systematic review was conducted and reported in accordance with Preferred Reporting Items for Systematic Reviews and Meta-Analyses (PRISMA 2020) guidelines, with synthesis performed in accordance with Synthesis Without Meta-Analysis (SWiM) recommendations. PubMed, Google Scholar, *ClinicalTrials.gov*, and ChiCTR were searched from January 2015 to August 2026. Risk of Bias (RoB) was assessed using study design-appropriate validated tools, including Cochrane RoB 2 and Risk of Bias In Non-randomized Studies - of Interventions (ROBINS-I) and methodological quality of uncontrolled studies was appraised using the Joanna Briggs Institute (JBI) Critical Appraisal Checklists for Case Series and Case Reports.

**Results:**

Sixteen publications comprising 18 patient cohorts across 12 neurological conditions were included. No treatment-related serious adverse events were reported. Most cohorts showed clinical improvement, while one reported stabilization, though the predominantly uncontrolled designs mean placebo response and natural disease history cannot be ruled out. However, the biologic class was strongly confounded by disease indication, dosing was heterogeneous and non-comparable, and five delivery techniques were used.

**Discussion:**

By integrating clinical evidence across biologic classes and neurological disorders, this review defines the current safety landscape, highlights major methodological gaps, and provides a framework for standardized future intranasal regenerative trials.

**Conclusion:**

Adequately powered, standardized, and controlled trials are required before clinical adoption can be considered.

## Introduction

Approximately 3.4 billion individuals worldwide suffer from neurological illnesses, which account for an overall estimated 443 million disability-adjusted life years (DALYs) yearly and are one of the biggest causes of global health loss (Collaborators, 2024). The mechanisms of neuronal injury, such as neuroinflammation, trophic deprivation, and impaired cellular repair, are not fully addressed by current pharmacological and rehabilitative approaches in neurodegenerative, neurodevelopmental, cerebrovascular, and uncommon neurological diseases (Collaborators, 2024). Growing interest in regenerative biologics, a diverse class of interventions that includes stem cells, extracellular vesicles (EVs) and exosomes, secretome and conditioned medium products, breast milk-derived biologic preparations, and related acellular products that are thought to work through overlapping principal mechanisms like paracrine signaling, immunomodulation, angiogenic support, and neuroprotective molecular cargo, has resulted from this ongoing therapeutic gap (Herman et al., 2021; Sun and Chang, 2024; Tang et al., 2024).

Since these interventions differ in composition, manufacturing process, and presumed mechanism, this review treats biologic class as a stratification variable rather than a basis for pooled comparison. Studies are grouped and reported by class at every analytic stage, including dosing, delivery method, and clinical outcome, and no claim of shared efficacy or a common mechanism across classes is made. What is compared across classes is procedural, namely, the feasibility and short-term tolerability of introducing a biologic preparation into the nasal cavity for central nervous system (CNS) access, independent of what that preparation contains. Mechanistic interpretation is addressed separately for each class where the evidence permits it.

The blood–brain barrier (BBB), which limits the accessibility of intravenously delivered drugs to the CNS and encourages peripheral biodistribution, is a significant obstacle to systemic biologic delivery in neurology (Wu et al., 2023). By taking advantage of the olfactory and trigeminal routes for direct nose-to-brain transport, intranasal administration has emerged as a potential means of circumventing this constraint (Giunchedi et al., 2020). Clinical translation remains challenging due to significant variations in nasal architecture, delivery device design, deposition-site targeting, dosage strategies, treatment plans, and product characterization standards, despite preclinical data supporting these considerations (Maumus et al., 2020). Human clinical evidence for intranasal regenerative biologics is now emerging across multiple neurological indications, with early-phase studies reporting feasibility and acceptable short-term safety profiles in conditions ranging from perinatal brain injury to neurodegenerative and neurodevelopmental diseases. This evidence remains dispersed across individual disease areas, biologic classes, and study designs, with no prior systematic synthesis covering the full scope of human clinical literature. The absence of a field-wide review limits the ability to assess the maturity of the current evidence, a critical step before definitive trials can be appropriately designed.

An International Prospective Register of Systematic Reviews-registered (PROSPERO) systematic review was conducted in accordance with Preferred Reporting Items for Systematic Reviews and Meta-Analyses (PRISMA 2020) reporting requirements, with narrative synthesis performed in compliance with Synthesis Without Meta-Analysis (SWiM) recommendations (Campbell et al., 2020; Page et al., 2021; Haby et al., 2022). This review includes human clinical studies investigating intranasal regenerative biologics for neurological disorders. Safety was the main quantitative synthesis anchor, with dose characterization, biologic class distribution, delivery method heterogeneity, disease-specific directional signals, and evidence maturity as determined by clinical development stage as secondary analytic domains.

## Materials and methodology

### Study design and reporting framework

The purpose of this systematic review was to compile the most recent human clinical data regarding the intranasal delivery of regenerative biologics, such as mesenchymal stromal cells (MSCs), neural stem cells (NSCs), EVs including exosomes, secretome and conditioned medium products, biologic preparations derived from breast milk, and related regenerative products meant for neural repair across neurological disorders. The primary analytic domain was safety, and secondary domains comprised dosing characteristics, biologic class distribution, intranasal delivery method, and condition-specific clinical response. Evidence maturity as determined by clinical development stage, trial phase, study design, and study-level validation characteristics across the included cohort. The review protocol was prospectively registered in PROSPERO (CRD420261381627) and predefined the review objectives, eligibility criteria, data extraction domains, RoB assessment approach, and synthesis strategy prior to data collection.

The review was conducted and reported in accordance with the PRISMA 2020 guidelines (Page et al., 2021). Given that the included studies were anticipated to be clinically and methodologically heterogeneous across neurological diseases, biologic classes, dosing approaches, delivery methods, and outcome measures, a quantitative efficacy meta-analysis was not performed because substantial heterogeneity precluded meaningful pooling of treatment effects. Synthesis without meta-analysis was therefore performed and reported in accordance with the SWiM recommendations (Campbell et al., 2020). Safety outcomes, specifically adverse events (AEs) and serious adverse events (SAEs), were selected as the primary quantitative synthesis endpoint because they represented the most consistently reported outcome domain across the included studies and were considered the most reliably extractable metric given the diversity of diseases, products, and study designs.

### Search strategy

A comprehensive systematic literature search was performed in PubMed, Google Scholar, and the clinical trial registries ClinicalTrials.gov and ChiCTR to identify human clinical studies on intranasal delivery of regenerative biologics in neurological disorders, published between January 1, 2015, and August 31, 2026. The search was restricted to January 2015 onward because literature on intranasal delivery of regenerative biologics in neurological disorders prior to this period was almost entirely preclinical, with no identifiable human clinical studies meeting the eligibility criteria of this review, and this date does not correspond to the endpoint of a prior systematic review. This review was scoped to CNS conditions for which biologics were delivered via the olfactory and trigeminal pathways and therefore excludes peripheral nervous system conditions such as peripheral neuropathies by design, rather than as a result of search strategy limitations. Clinical trial registries were searched to identify additional eligible studies and to supplement publication data. Registry-only entries without an associated peer-reviewed publication were not included as independent evidence sources. The search was limited to English-language publications due to practical resource constraints and the predominance of English in biomedical publishing. Reference lists of all full-text-assessed studies were also hand-searched to identify any additional relevant records.

Search terms were grouped into three blocks, combined with AND. The first block covered delivery route and included: “intranasal,” “transnasal,” “nasal administration,” “nasal delivery,” “nose-to-brain,” and “olfactory delivery.” The second block covered intervention type and included: “stem cell,” “mesenchymal stem cells,” “mesenchymal stromal cells,” “neural stem cell,” “extracellular vesicles,” “exosomes,” “secretome,” “conditioned medium,” “conditioned media,” “breast milk,” “human milk,” “cell therapy,” AND “regenerative biologics.” The third block covered neurological indication and included broad anchor terms “central nervous system,” “brain,” “neurodegenerative,” AND “neurodevelopmental” alongside specific disease terms: “Alzheimer disease,” “Alzheimer’s disease,” “Parkinson disease,” “Parkinson’s disease,” “amyotrophic lateral sclerosis,” “autism spectrum disorder,” “cerebral palsy,” “stroke,” “perinatal stroke,” “intraventricular hemorrhage,” “dementia”, Broad neurological terms were used to ensure that conditions not individually named were not missed. Human study filters available in PubMed were applied rather than Boolean NOT exclusions, to avoid accidently removing human papers that mention animal studies in passing. Registry records without a linked published report were not included as standalone evidence but were used to identify associated publications. Full database-specific search strings, including Medical Subject Headings (MeSH) terms for PubMed, are provided in Supplementary Appendix A1.

### Eligibility criteria

Studies were eligible if they reported intranasal delivery of regenerative biologics in human patients with a neurological disorder. Eligible biologic classes included MSCs, NSCs, iPSC-derived products, EVs, exosomes, secretome, conditioned medium, and breast milk-derived preparations. Eligible studies were limited to those published in English. At least one patient-level safety, clinical, functional, imaging, feasibility, or dosing outcome was required for inclusion. Any comparator was acceptable, including placebo, standard of care, historical control, and no comparator. Eligible study designs included randomized control trial (RCT), phase 1, phase 1/2, and phase 2 trials, non-randomized prospective studies, open-label studies, case series, and single-patient case reports. A total of 95 studies were excluded for using non-intranasal routes, involved only drug interventions, or reported no patient-level outcomes. Preclinical, animal-only, in vitro, and review publications were excluded. Conference abstracts without patient-level primary data were excluded, and those reporting patient-level primary data were included. No study was excluded solely based on comparator type or absence thereof. Full inclusion and exclusion criteria are provided in Table 1.

**TABLE 1:** PICS eligibility criteria.

PICOS	Included	Excluded
Population (P)	Human patients of any age with a diagnosed neurological disorder Includes preterm neonates, children, adults, and elderly patients Conditions covered: • Neurodegenerative disorders • Neurodevelopmental disorders • Cerebrovascular diseases • Perinatal brain injury • Rare neurogenetic disorders	• Animal studies • In vitro, ex vivo, or cell line studies without human patient data • Healthy volunteers without a neurological diagnosis
Intervention (I)	Intranasal regenerative biologics, including: • MSCs • NSCs • iPSC-derived cells and iPSC-derived EVs • EVs and exosomes • Secretome and conditioned medium • Breast milk-derived biologic preparations used in neonatal neuroprotection studies • Related regenerative biologic preparations intended for neural repair	• Non-intranasal routes of administration • Non-stem cell-based treatments • Drug-only interventions • Combined drug and biologic studies where the intranasal regenerative biologic component could not be separately identified or quantified
Comparator (C)	Any comparator or none. Studies with no comparator arm were eligible Acceptable comparators included placebo, sham, standard of care, historical control, and concurrent untreated control	No study was excluded on the basis of comparator type or absence
Outcomes (O)	Primary outcome: Safety, including AEs, SAEs, treatment-related AEs, treatment-related SAEs, deaths, and withdrawals due to AEs Secondary outcomes: • Disease-specific clinical and functional outcomes • Neurodevelopmental outcomes • Imaging outcomes • Feasibility outcomes • Dosing characteristics At least one patient-level outcome was required for inclusion	• Study protocols without outcome data • Conference abstracts without patient-level primary data
Study Design (S)	• RCTs • Phase 1, phase 1/2, and phase 2 trials • Non-randomized prospective studies • Open-label studies • Case series • Single-patient case reports Case reports were eligible because for several conditions in this review, single-patient reports represent the only available human clinical evidence Conference abstracts reporting patient-level primary data were included	• Preclinical studies • Animal-only studies • In vitro studies • Reviews, protocols, editorials, and commentaries • Conference abstracts without patient-level primary data • Reports in which no patient received intranasal therapy

Abbreviations: AE, adverse event; EVs, extracellular vesicles; iPSC, induced pluripotent stem cell; MSCs, mesenchymal stem cells; NSCs, neural stem cells; RCT, randomized controlled trial; SAE, serious adverse event.

### Data extraction

For each eligible study, standardized data extraction was performed by two independent reviewers (UEH and IS) extracted study identification details, year of publication, country, clinical setting, trial registration number, study design, neurological condition, sample size, comparator type, participant age group, participant characteristics, biologic class, product source, route of administration, intranasal delivery method, dose, dosing unit, number of administrations, treatment interval, cumulative intranasal exposure, follow-up duration, primary clinical outcome, direction of clinical effect, AEs, SAEs, treatment-related SAEs, deaths. In cases where discrepancies arose during the study selection process, a third reviewer (DLG) was consulted.

Dose was extracted in the units reported by each study, including cells/kg, particles, micrograms, milliliters, or study-specific units. Cumulative intranasal exposure was calculated using the equation:

Cumulative Dose=Dose Per Administration×Number of Administrations

Cross-product quantitative dose comparisons were not attempted due to the heterogeneity of dose units across biologic classes. For studies involving multiple routes of administration, the intranasal component was extracted separately where possible. The systemic component was documented for context but was not included in dose characterization. The remaining data was also collected for the in-house repository. Access to this repository, is available on reasonable request to the corresponding author, who will provide the link.

Clinical outcomes were extracted using the primary outcome measure designated by the study authors or, where no primary outcome was explicitly specified, the principal efficacy endpoint. Outcomes included cognitive scales, motor scales, functional scales, neurodevelopmental scales, autism-related scales, Amyotrophic Lateral Sclerosis (ALS) functional scales, cerebral palsy functional classifications, and neuroimaging outcomes. These outcome measures are not directly comparable across diseases, and clinical efficacy was not pooled statistically. The direction of effect was determined from the primary outcome reported by the study investigators and categorized as improvement, stability, deterioration, or insufficient information. Where p-values were available, statistical significance (p < 0.05) was used as the threshold for determining a positive or negative direction. For studies without inferential statistics, predominantly case reports and small case series, the direction was based on the magnitude and consistency of change from baseline as reported by the authors. Studies reporting only safety or feasibility outcomes, with no efficacy endpoint, were classified as insufficient information for directional synthesis. Safety outcomes were extracted as AEs, SAEs, treatment-related AEs and SAEs, deaths, and adverse event-related withdrawals. Safety data were verified against the primary publication and, where available, trial registry records. No discrepancies between sources were recorded.

### Handling of missing and unclear data

When data were missing from a published report, the corresponding trial registry record was checked first. If the information was still unavailable, the corresponding author was contacted by email up to 2 times, with at least 2 weeks between attempts. Six studies (Boika et al. (2020); Sonmez Demir et al. (2025); Gesundheit et al. (2025); Sinelnyk et al. (2024), Ueda and Seta (2022), Ekici et al. (2026)), in this review did not have a trial registry number on record. All variables for which information could not be obtained were recorded as not reported. Nothing was assumed, and nothing was imputed. This same approach was applied to all variables, regardless of whether the missing information concerned dose, safety outcomes, follow-up duration, or clinical results.

Specifically for dose, one eligible study by Sinelynk et al. (2024) did not report sufficient information to calculate cumulative intranasal exposure. The corresponding author was contacted by email on two separate occasions, with at least 1 week between each attempt, but no response was received (Sinelnyk et al., 2024). This study was included in qualitative synthesis, safety analysis, disease mapping, and delivery method analysis, but was excluded from dose characterization. No other variables required exclusion from predefined analyses because missing information was limited and handled descriptively.

### Risk of bias assessment

The RoB was assessed independently by two reviewers (UEH and IS) for each included study, using a tool selected according to study design. Disagreements were resolved through discussion and, where necessary, by referral to a third reviewer (SS). The RCT was assessed using the Cochrane RoB 2 tool (Sterne et al., 2019). Non-randomized comparative studies with a control arm were assessed using the Risk Of Bias In Non-randomized Studies - of Interventions (ROBINS-I) tool. For early-phase open-label, before–and–after, or single-arm studies, where no parallel comparator group was available, ROBINS-I domains were not interpreted as formal comparative causal judgments. Instead, these studies were appraised with attention to methodological limitations such as participant selection, intervention reporting, outcome measures, follow-up completeness, and selective reporting.

Findings from these studies were therefore interpreted primarily as feasibility, safety, and preliminary efficacy signals rather than definitive evidence of comparative treatment effect (Sterne et al., 2016). Uncontrolled studies enrolling multiple participants were assessed using the Joanna Briggs Institute (JBI) Critical Appraisal Checklist for Case Series (Munn et al., 2020), and single-patient case reports were assessed using the JBI Critical Appraisal Checklist for Case Reports (Munn et al., 2020). For both JBI checklists, each item was rated as ‘Yes, Unclear, No, and Not Applicable’. No summary score or overall risk classification was assigned, consistent with JBI guidance that these tools are intended to inform appraisal rather than produce a single composite judgment. As the included literature spanned multiple study designs across 12 neurological conditions, the RoB was not collapsed into a single numerical score. Bias profiles were summarized narratively by study design group, following the judgment criteria appropriate to the scale.

Case series and case reports were retained specifically for field-mapping and hypothesis-generation purposes given the limited body of controlled evidence for several of the 12 conditions covered. Consistent with their appraisal tier above, findings from these designs were never pooled quantitatively with those from the RCT or the non-randomized comparative studies.

## Results

### Study selection

The search across PubMed, Google Scholar, ClinicalTrials.gov, and ChiCTR returned 18,670 records in total, with 18,641 from databases and 29 from registries, plus one additional record identified through citation searching. All records retrieved from PubMed, Google Scholar, ClinicalTrials.gov, and ChiCTR were exported to a reference-management database and merged into a single library. Duplicate records were identified through automated matching of bibliographic fields, including DOI, PMID, and trial registration numbers. Potential duplicates were then checked manually to ensure that multiple records referring to the same study or publication were removed only once. None of the included studies relied solely on registry records; each study had a linked published report. The full selection process is illustrated in the PRISMA flow diagram (Figure 1).

**FIGURE 1:**
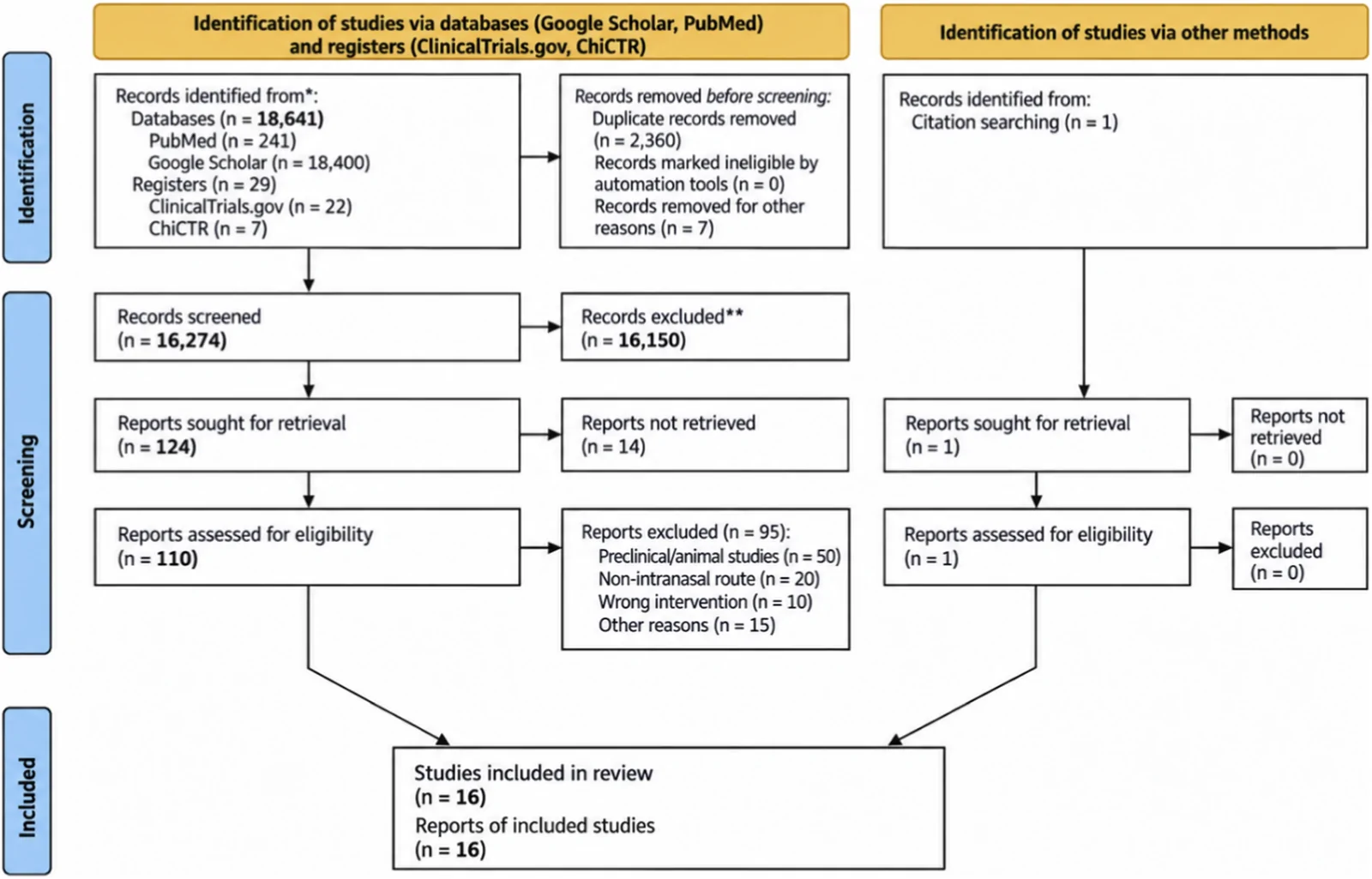
PRISMA Flow Diagram illustrating identification, screening, and selection of the eligible clinical trials/studies for systematic review.

The 19 reports came from 16 unique publications and covered 18 distinct patient cohorts. Two of the studies Baak et al. (2022) and Wagenaar et al. (2025), described the same PASSIoN cohort of 10 patients at 3- and 24-month follow-up points, so they were counted as one publication and one cohort (Baak et al., 2022; Wagenaar et al., 2025). One publication reported four different disease cohorts within the same paper. Prodromos et al. (2025) included separate patient groups for ALS, Lewy body dementia (LBD), Kennedy disease (KD), and congenital myasthenic syndrome (CMS). Therefore, although it was counted as one publication, it contributed four separate disease-specific reports and four cohorts to the review (Prodromos et al., 2025). The remaining 14 publications, including Tarjanyi et al. (2026), each contributed one report and one cohort. Taken together, the 18 cohorts spanned 12 neurological conditions.

### Study characteristics

The 16 included publications covered 18 patient cohorts spread across 12 neurological conditions. Study designs were mixed, running from a phase 1/2 RCT and early-phase open-label trials at one end to case series and single-patient reports at the other. The available clinical evidence comprised 4 phase 1 trials, 2 phase 1/2 trials, 4 case series, 5 case reports, and 4 other uncontrolled or non-randomized studies. The other 4 studies included Morita et al. (2024) single-arm open-label, Boika et al. (2020) open-label non-randomized, Wagenaar et al. (2025) post hoc 2-year follow-up, and Sonmez Demir et al. (2025) prospective non-RCT. Six biologic classes appeared across the cohorts, with EVs, including exosomes, coming up most often. The mode of delivery of biologics, units of measurement for the doses and follow-up times of the participants varied considerably from study to study. Total enrollment per study ranged from 1 to 228 participants. The number of patients who received intranasal treatment ranged from 1 to 37. The largest enrolled study had a substantial retrospective control group. Follow-ups ranged from around 4 weeks to 39 months. Study-level characteristics, including enrollment size, treated sample size, intervention type, cumulative dosing, and follow-up duration, are summarized in Table 2.

**TABLE 2:** Study characteristics of included studies.

Disease	Study		Population			Intervention		Cumulative dose/administration schedule	Key outcomes	F/U (mo)
	Registry no./References	Study design	n (Tx/Ctrl)	Sex (M/F)	Age	Biologic class	Delivery route			
AD	/NCT04388982 (Xie et al., 2023)	Phase I/II	9 (9/0)	4/5	68.3 years	ahaMSC-Exosomes	Nasal spray	0.48–1.92 × 10^10^ particles. NR	ADAS-cog ↓2.33; MoCA-B ↑2.38	12
	UMIN000053551 (Morita et al., 2024)	Single-arm open-label	13 (13/0)	6/7	77.8 years	AD-MSC secretome	Nasal spray	24 vials. NR	HDS-R 15.6→17.5 (p = 0.030)	2
PD	ChiCTR1900024496 (Jiang et al., 2024)	Phase 1 dose-escalation	18 (18/0)	13/5	64.1 yr	hNSCs	Neuroendoscope	6–60 × 10^6^ cells. NR	MDS-UPDRS ↓19.9 at 6 months	6
	(Boika et al., 2020)	Open-label non-randomized	6 (6/0)‡	NR/NR	58.7 years	Autologous BM-MSC	IN + IV (tandem)‡	0.7–8.97 × 10^6^ cells (IN). NR	UPDRS-III OFF 36.5→33.5 (p = 0.02)	3
PAIS	NCT03356821 (Baak et al., 2022)	Phase 1 open-label	10 (10/0)	4/6	6.1 d postnatal	Allogeneic BM-MSC	Catheter droplets	45–50 × 10^6^ cells. NR	1 transient mild fever; no SAEs	3
	NCT03356821 (Wagenaar et al., 2025)	Post-hoc 2years follow-up	10 (10/0)	4/6	6.1 d postnatal	Allogeneic BM-MSC	Catheter droplets	45–50 × 10^6^ cells. NR	CP 20% vs. 49%; motor Z 0.27 vs. −0.42 (p = 0.003)	24
GM-IVH	(Sonmez Demir et al., 2025)	Prospective non-RCT	22 (11/11)	6/16	27.6 wk GA	Fresh breast milk	Insulin syringe drop	∼11–22 mL (≥28days). NR	PHVD 27.3% vs. 81.8% (p = 0.010)	30
	NCT04225286 (Hoban et al., 2024)	Phase 1 feasibility/safety	37 (37/0)	22/15	27.3 wk GA	Fresh parent’s own milk	Oral syringe/nasal vestibule	∼22.4 mL (28days) NR	Feasibility/safety: no IHM-related SAEs	1
	NCT04225286 (Gallipoli et al., 2025)	Phase 1 non-RCT + historical ctrl	228 (37/191)	113/115	27.3 wk GA	Fresh parent’s own milk	Oral syringe/nasal vestibule	∼22.4 mL (28days) NR	Fine motor delay OR 0.35 (p = 0.03)	18
ASD	(Gesundheit et al., 2025)	Case report	1 (1/0)	1/0	7.5 years	Allogeneic MSC-exosomes	IN exosomes‡	∼2.6 × 10^10^ exosomes. Escalated (2→60 admins)	ATEC 149→11; SRS-2 193→32	39
	(Sinelnyk et al., 2024)	Case report	1 (1/0)	1/0	12 years	Fetal NSCs + FSC-exosomes	IN + SC + inhalation + IV‡	NR. 2-day course	ATEC 60→28	12
CP	NCT03005249 (Lv et al., 2023)	Phase 1/2 RCT	25 (15/10)	17/7	6.3 years	Fetal forebrain NSC	Olfactory-fissure patch	1.5 × 10^6^ cells/kg. Monthly	GMFM-88 75.0 vs. 41.9 (p = 0.009)	24
ALS	(Ueda and Seta, 2022)	NR Case report	1 (1/0)	1/0	68 years	SHED-CM	Transnasal‡	NR. Weekly	ALSFRS-R stable 12/48; ↑ROM	12
	NCT07105371 (Prodromos et al., 2025)	Open-label case series†	14 (14/0)	13/1	55.7 years	UC-MSC exosomes (AlloEx)	Bilateral IN (Tx360® applicator)	∼1.6 × 10^12^–1 × 10^13^ particles Every 2 months	ALSFRS-R 30.93→34.00; 13/14 improved	6
LBD	NCT07105371 (Prodromos et al., 2025)	Case series†	2 (2/0)	2/0	68.5 years	UC-MSC exosomes (AlloEx)	Bilateral IN (Tx360® applicator)	Pt1 ∼4 × 10^12^; Pt2 ∼2.8 × 10^12^ 2–3 months intervals	Pt17 cognition ↑80%, motor ↑60%	6
KD	NCT07105371 (Prodromos et al., 2025)	Case report†	1 (1/0)	1/0	66 years	UC-MSC exosomes (AlloEx)	Bilateral IN (Tx360® applicator)	∼1.6 × 10^12^ particles. 2 consecutive days	Pain 8/10→1–2/10; fasciculations ↑50%	2
CMS	NCT07105371 (Prodromos et al., 2025)	Case report†	1 (1/0)	0/1	47 years	UC-MSC exosomes (AlloEx)	Bilateral IN (Tx360®)‡	∼2 × 10^12^ particles 2 consecutive days	MIP 26→73; grip L 35→50, R 35→60 lbs	1.75
CSS	(Ekici et al., 2026)	Retrospective case series	3 (3/0)	2/1	6.4 years	hUC-MSC exosomes	Nasal spray	300–600 µg protein. Monthly	Vineland-3 ABC 83.0→90.7 (+9%)	1.5
HIE	NCT06747260 (Tarjanyi et al., 2026)	Prospective feasibility study	10 (10/0)	4/6	1.0 days postnatal	Own-mother’s fresh breast milk	Intranasal drops (BID)	∼40 mL (from 48h after birth to 28days) Twice daily	No AEs; 100% initiated ≤48h; 1 death (unrelated)	1

Abbreviations: AD, Alzheimer’s disease; ADAS-cog, Alzheimer’s Disease Assessment Scale–Cognitive Subscale; AD-MSC, adipose-derived mesenchymal stromal cell; ABC, aberrant behavior checklist; ahaMSC-Exo, allogeneic human adipose mesenchymal stromal cell-derived exosomes; AlloEx, umbilical cord-derived MSC, exosome product (Vitro Biopharma); ALS, amyotrophic lateral sclerosis; ALSFRS-R, Amyotrophic Lateral Sclerosis Functional Rating Scale–Revised; ASD, autism spectrum disorder; ATEC, autism treatment evaluation checklist; BID, twice daily; BM-MSC, bone marrow-derived mesenchymal stromal cell; CMS, congenital myasthenic syndrome (DOK7); CP, cerebral palsy; Ctrl, control group; CSS, Coffin-Siris syndrome; FSC, fetal stem cell; F/U, follow-up; GA, gestational age; GMFM-88, Gross Motor Function Measure–88; GM-IVH, germinal matrix–intraventricular hemorrhage; HDS-R, revised hasegawa dementia scale; HIE, hypoxic-ischemic encephalopathy; hNSC, human neural stem cell; IHM, intranasal human milk; IN, intranasal; IV, intravenous; KD, kennedy disease; LBD, lewy body dementia; MDS-UPDRS, Movement Disorder Society–Unified Parkinson’s Disease Rating Scale; MIP, maximal inspiratory pressure; MoCA-B, Montreal Cognitive Assessment–Basic; mo, months; MSC, mesenchymal stromal cell; n, total sample size; NR, not reported; NSC, neural stem cell; NST, non-stress test; OR, odds ratio; PAIS, perinatal arterial ischemic stroke; PD, Parkinson’s disease; PHVD, post-hemorrhagic ventricular dilatation; ROM, range of motion; SAE, serious adverse event; SC, subcutaneous; SHED-CM, stem cells from human exfoliated deciduous teeth-conditioned medium; SRS-2, social responsiveness scale, Second Edition; Tx, treatment group; UC-MSC, umbilical cord-derived mesenchymal stromal cell; UPDRS-III, Unified Parkinson’s Disease Rating Scale Part III; h, hour(s); d, day(s); wk, week; yr, years.

† Prodromos et al. (2025) (NCT07105371) is presented as four rows (ALS, LBD, KD, CMS) for clarity but represents one publication throughout.

‡ Mixed-route study; only the intranasal component is reported here. Doses were reported in cells, cells per kilogram body weight, particles, micrograms of protein, milliliters, percentage concentrations, and vial count without per-vial characterization. For Morita et al. (2024), the cumulative dose is given as 24 vials because per-vial cell number, protein content, and volume were not reported, so an equivalent dose in comparable units cannot be derived.

### Risk of bias assessment

#### Cochrane RoB 2 tool

Lv et al. (2023) (NCT03005249), the only RCT in this review, was assessed with the Cochrane RoB 2 tool against the 24-month Gross Motor Function Measure-88 (GMFM-88) and Activities of Daily Living (ADL) outcomes. Two of the five domains raised ‘some concerns’. Even though baseline groups were uniform, randomization raised ‘some concerns’. The report confirmed random assignment but provided no details on sequence generation or allocation concealment. Deviations from intended interventions were also a concern, since the open-label design left patients and clinicians aware of allocation. Both arms nonetheless followed the same rehabilitation protocol, and outcome assessors stayed blinded.

The other three domains were ‘low’ risk. Follow-up was near complete, with 24 of 25 patients retained at 24 months, and the one dropout was attributable to a COVID-19 lockdown. The outcome measures were validated instruments for pediatric cerebral palsy (CP), scored by blinded assessors, and the reported endpoints matched those in the December 2016 prospective registration. Overall results of RoB showed ‘some concerns’ driven by incomplete randomization reporting rather than any observed bias (Figure 2A).

**FIGURE 2:**
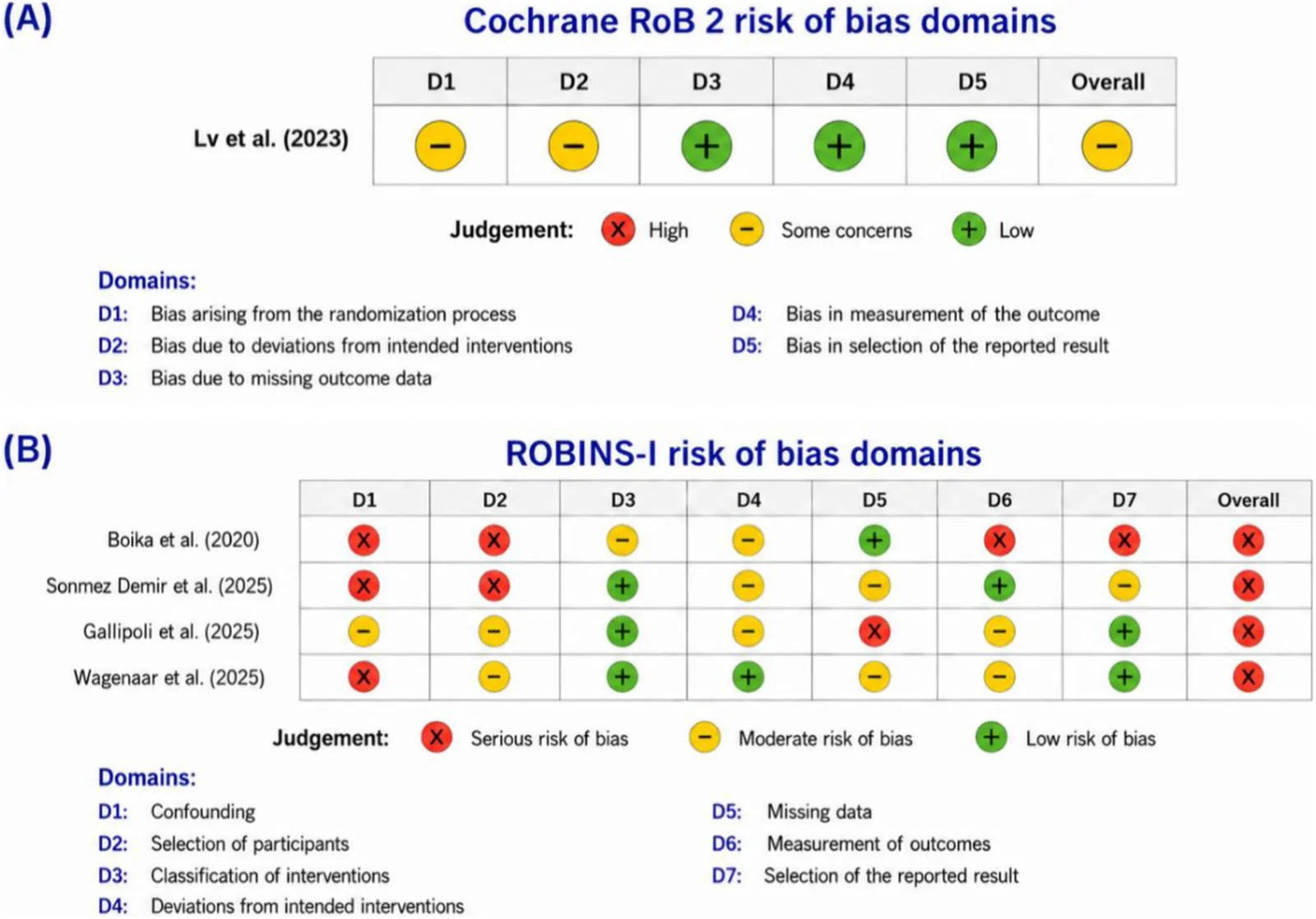
**(A)** RoB assessment for the RCT using the Cochrane RoB 2 tool across the five domains and the overall judgment. D1, randomization process; D2, deviations from intended interventions; D3, missing outcome data; D4, measurement of the outcome; D5, selection of the reported result. **(B)** RoB assessment for the four non-randomized studies using the ROBINS-I tool across the seven domains and the overall judgment. D1, confounding; D2, selection of participants; D3, classification of interventions; D4, deviations from intended interventions; D5, missing data; D6, measurement of outcomes; D7, selection of the reported result.

#### ROBINS-I assessment of non-randomized comparative studies (n-RCTs)

The ROBINS-I tool was applied to four (Boika et al., 2020; Sonmez Demir et al., 2025; Gallipoli et al., 2025; Wagenaar et al., 2025) n-RCTs across seven domains. All four received an overall judgment of ‘serious RoB’. Under ROBINS-I, a serious judgment in any single domain determines the overall rating regardless of performance in other domains. Domain-level judgments are presented in Figure 2B.

Boika et al. (2020) were rated ‘serious’ across four domains. Treatment assignment based on individual clinical judgment, without protocol or statistical adjustment, led to serious ratings for Domain 1 (confounding) and Domain 2 (participant selection). Domain 6 (measurement of outcomes) was rated as ‘serious’ because endpoints, including the Unified Parkinson’s Disease Rating Scale Part III (UPDRS-III) and Parkinson’s Disease Questionnaire-39 (PDQ-39), were collected without assessor blinding in an open-label trial, and the authors acknowledged a risk of placebo effect. The absence of trial registration meant selective outcome reporting could not be excluded, resulting in a ‘serious’ rating for Domain 7 (selection of the reported result). Sonmez Demir et al. (2025) were rated ‘serious’ for Domain 1 (confounding) and Domain 2 (selection of participants) because groups formed spontaneously based on maternal proximity to the neonatal intensive care unit (NICU) and ability to supply fresh breast milk, introducing socioeconomic confounding that could not be adjusted post hoc. Domain 6 (measurement of outcomes) was rated ‘low RoB’ because the primary ultrasound outcome was graded by a radiologist explicitly blinded to allocation status.

Gallipoli et al. (2025) achieved a ‘moderate RoB’ rating for Domain 1 (confounding) through the application of inverse probability weighting (IPW) with G-computation, adjusting for intraventricular hemorrhage (IVH) grade, gestational age, and sex. Under ROBINS-I, a ‘serious RoB’ judgment in any domain results in an overall ‘serious RoB’ classification. The overall ‘serious’ rating was driven entirely by Domain 5 (missing data), with up to 78% missing data for some outcomes, skewed towards the historic control cohort during pandemic-era restrictions on in-person developmental testing.

Wagenaar et al. (2025) performed well across most domains, with ‘low RoB’ ratings for Domain 3 (classification of interventions), Domain 4 (deviations from intended interventions), and Domain 7 (selection of the reported result), the last of which was supported by transparent post hoc framing of the registry comparison. Domain 1 (confounding) was rated ‘serious RoB’ because the historic registry comparator spanned birth years 1994–2022 without statistical adjustment for temporal changes in NICU care practices over that period. Across all four studies, ‘serious RoB’ was most consistently driven by non-randomized treatment allocation, absence of assessor blinding, and use of historical comparators without statistical correction.

### JBI case series appraisal

The JBI Critical Appraisal Checklist for Case Series covers ten items, referred to throughout as items Q1 to Q10. Each item is rated ‘Yes, No, Unclear, or Not Applicable’. Findings are reported at the item level (Figure 3A). Two reviewers (UEH and IS) independently appraised each study. Disagreements were resolved by consensus.

**FIGURE 3:**
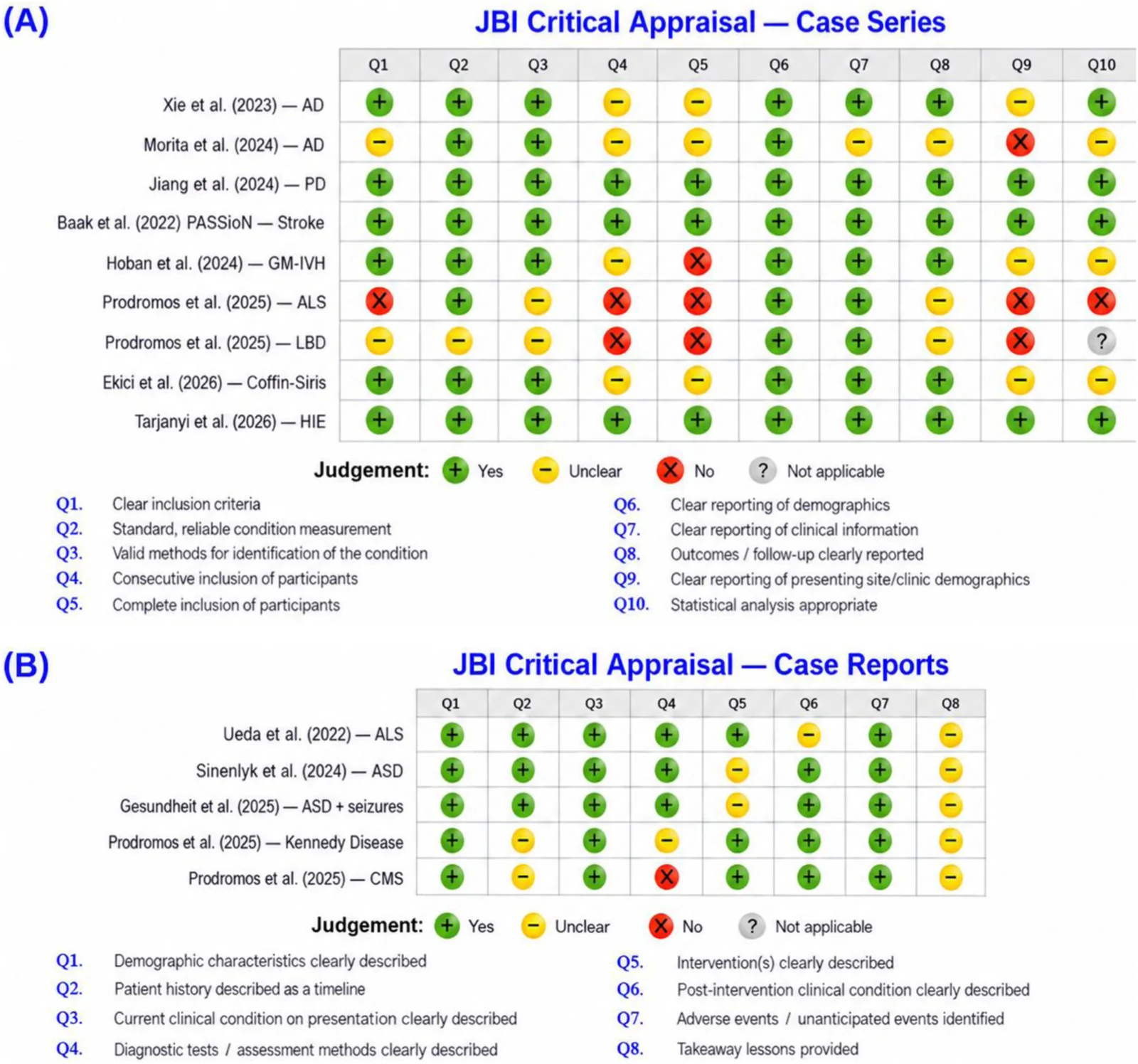
JBI item-level appraisal of case series and case reports. **(A)** Case series, appraised against the ten items (Q1 to Q10) of the JBI Critical Appraisal Checklist for Case Series. **(B)** Case reports, appraised against the eight items (Q1 to Q8) of the JBI Critical Appraisal Checklist for Case Reports. Each item was rated ‘Yes’, ‘No’, ‘Unclear’ or ‘Not Applicable’ by two independent reviewers, with disagreements resolved by consensus. Neither checklist yields a summary score, so results are presented at the item level.

Jiang et al. (2024), Baak et al. (2022), PASSIoN, and Tarjanyi et al. (2026), F-NEO-BRIGHT received ‘Yes’ on all ten items. All three had clearly defined inclusion and exclusion criteria, used validated diagnostic and outcome measures, and enrolled participants consecutively with a fully reported screening-to-enrollment flow. Site demographics were complete across all three, and the statistical methods (descriptive statistics for feasibility and safety endpoints) matched each study’s design. Xie et al. (2023), Hoban et al. (2024), and Ekici et al. (2026) were rated ‘Unclear or No’ on Q4 (consecutive inclusion) and Q5 (complete inclusion). None of the three reported a screening-to-enrollment flow, so it is not possible to tell how many eligible patients were excluded or why. Hoban et al. received a ‘No’ on Q5. Only 37 of 108 eligible infants were enrolled, after a mid-study protocol amendment changed the inclusion criteria partway through. Morita et al. (2024) had the most ‘Unclear’ ratings among the Alzheimer’s disease studies: Q1, Q4, Q5, Q7, Q8, and Q10. It received a ‘No’ on Q9. Site demographics were not reported. The analysis was descriptive, across six participants. With only six patients and no statistical testing, the finding could easily reflect chance rather than a true effect.

The two Prodromos et al. (2025) sub-cohorts had the most ‘No’ ratings in the panel. Patients were self-referred through a private clinic. This pathway, rather than a defined referral or screening process, drove the ‘No’ ratings on Q4 and Q5. Site demographics were not reported (Q9, ‘No’). Outcome reporting mixed validated instruments with unvalidated Likert scales (Q8, ‘Unclear’). When two different types of measurement are combined this way, the results from different patients are not comparable. Across the case series overall, the same gaps recurred, including incomplete reporting of patient selection, missing screening-flow data, and analysis limited to descriptive statistics. Intervention and follow-up reporting were the exception. Most studies described these clearly, regardless of how they scored elsewhere.

### JBI case reports appraisal

The JBI Critical Appraisal Checklist for Case Reports rates eight items (Q1-Q8) as ‘Yes, No, Unclear, or Not Applicable’ and gives no summary score, so results are reported at the item level (Figure 3B). Two reviewers (IS, UEH) appraised each full text independently and settled disagreements by consensus. Five single-patient rows from four publications were assessed.

Gesundheit et al. (2025) was the only report with ‘Yes’ on all eight items. Diagnosis was based on karyotype and sequence analyses by independent assessors. The outcomes were tracked with Autism Diagnostic Observation Schedule (ADOS), Autism Treatment Evaluation Checklist (ATEC), Social Responsiveness Scale Second Edition (SRS-2), and serial Electroencephalography (EEG), and dosing was recorded for all five sessions with GMP-level detail, including a mild fever after session three that prompted a dose reduction. The remaining reports carried isolated ‘Unclear’ ratings. Ueda and Seta (2022) was ‘Unclear’ on Q7 because a tracheostomy was attributed to ALS progression without a formal causality assessment. Sinelnyk et al. (2024) was ‘Unclear’ on Q5, since dosing was not given per route in a multi-route protocol, and on Q8, since the 2-month follow-up was too short to judge durability. Prodromos et al. (2025) KD was ‘Unclear’ on Q2 and Q4, the latter because diagnosis lacked androgen-receptor CAG-repeat confirmation, the standard test for this X-linked disorder. These are reporting gaps that affect interpretability, not flaws in how the studies were run. Prodromos et al. (2025) CMS was the only report with a ‘No’ on Q4. The diagnosis was physician-confirmed but had no genetic sequencing, repetitive nerve stimulation, or single-fiber electromyography, which are standard for a neuromuscular condition with 35 known causative genes. A concurrent intravenous MSC infusion on day three could not be separated from the intranasal component in the outcome data, so effects cannot be attributed to the intranasal route alone. This issue sits outside the JBI checklist. Across the five rows, the recurring concerns were diagnostic confirmation gaps in rare neuromuscular conditions and incomplete attribution in mixed-route protocols, while detailed outcome tracking and clear intervention reporting were the consistent strengths.

### Stratified safety synthesis

All 18 patient groups contributed their own follow-up window, which ranged from as little as 1 month in the hypoxic-ischemic encephalopathy (HIE) cohort (Tarjanyi et al., 2026) to as long as 39 months in the Gesundheit et al. (2025) Autism spectrum disorder (ASD) case, and investigators determined that no SAE was connected to intranasal treatment. Although the cumulative exposed population is still small and safety surveillance varies significantly in duration and intensity between cohorts, the results were comparable across all six biologic classes, all neurological diseases, and all administration systems. Therefore, rather than being conclusive safety data, these results should be interpreted as early feasibility and tolerability signs.

When adverse effects were reported, they were generally short-lived and mild in severity. The most commonly described symptoms were fever, shaking, headache, transient nasal irritation, and pain at the application site. However, adverse-event reporting was limited in some studies, particularly Boika et al. (2020) and Morita et al. (2024), so the apparent absence of complications in these cohorts should be interpreted cautiously. Overall, thirteen cohorts reported no adverse events, supporting a generally favorable short-term safety profile, although stronger and more standardized safety reporting is still needed. Jiang et al. (2024) reported transient olfactory irritation, headache, and discomfort at the application site across the three dose cohorts. These events were considered by the investigators to be related to the administration procedure rather than the cell product itself, and no dose-limiting toxicity was observed. Baak et al. (2022) described one brief episode of fever in a neonate after intranasal MSC administration. Similarly, Gesundheit et al. (2025) reported a short episode of fever and shivering after the third treatment session. The symptoms resolved spontaneously, although the dose was reduced for the following session as a precaution. Lv et al. (2023) also noted one mild episode of shivering, that had settled without any intervention. The most clinically complex case was reported by Ueda and Seta (2022), where tracheostomy and aspiration pneumonia occurred during follow-up. The investigators assign these events to ALS progression rather than the treatment itself. However, because no formal framework for causality assessment was described, this attribution should be interpreted with caution. Given the predominance of early-phase, uncontrolled, and small-sample studies, together with heterogeneous adverse-event reporting across cohorts, these observations are best understood as preliminary feasibility and tolerability signals rather than definitive safety evidence.

### Dose distribution across biologics

Cumulative intranasal doses varied substantially across biologic classes and could not be placed on a common axis. A formal cross-study dose comparison or dose-response analysis was therefore not possible. These distributions for whole-cell and exosome/EV studies, shown on logarithmic scales, are presented in Supplementary Figure S1 to describe dose reporting. Exosome and EV doses spanned more than three orders of magnitude within this single biologic class.

Doses were given in cells, cells per kilogram body weight, particles, micrograms of protein, milliliters, percentage concentrations, and vial count without per-vial characterization. Six cohorts could not be placed on either dose axis for this reason. Morita et al. (2024) reported vial counts without per-vial mass or particle data. Sonmez Demir et al. (2025), Hoban et al. (2024), and Gallipoli et al. (2025) reported milliliter volumes of breast milk or parent’s own milk without cellular or particle characterization. Ueda and Seta (2022) reported a percentage concentration without volume, and Ekici et al. (2026) reported micrograms of protein only. Sinelnyk et al. (2024), reported no dose data at all. These seven cohorts make up a substantial share of the reviewed literature that could not be characterized on a dose axis.

The four Prodromos et al. (2025) sub-cohorts are the only within-product cross-disease comparison in this dataset where the same commercially characterized UC-MSC exosome preparation and administration protocol was used across ALS, LBD, KD, and CMS. In the remaining cohorts, biologic class selection was confounded with disease indication, so any observed differences could reflect disease, product, or dose effects, and there is no way to separate the three. Given this variation in product type, dose unit, delivery method, administration schedule, and patient population, the dataset does not support a reliable dose-response assessment for any biologic class.

### Comparative evidence across biologic classes

Six biologic classes were identified across 12 neurological conditions in the 18 included cohorts, with the distribution shown in Table 3. Biologic class selection was strongly driven by disease indication throughout the studies. No condition was studied with more than one biologic class in a comparative framework, which means it is not possible to disentangle whether observed clinical patterns reflect the biologic itself, the disease, or both. The included studies are unevenly distributed, with most evidence arising from only a few conditions and treatment categories. Replicated within-condition evidence exists for only three conditions that are Alzheimer’s disease (AD), Parkinson’s disease (PD), and germinal matrix-intraventricular hemorrhage (GM-IVH) in preterm neonates. Every other condition is represented by a single cohort or individual case report. The apparent use of exosomes and EVs across multiple conditions is largely attributable to one publication reporting the same product in four disease sub-cohorts, rather than independent replication by separate research groups. These structural features mean that disease and biologic class confounding remains a central limitation of this reviewed literature and cannot be resolved through synthesis alone.

**TABLE 3:** Distribution of intranasally treated patients by neurological condition, biologic class, and delivery technique across the 18 included cohorts.

Disease	Biologic classes	Delivery technique(s)	Cohorts (k)	Patients treated (n)
AD	ahaMSC-exosomes; AD-MSC secretome	Nasal spray	2	22
PD	hNSCs; Autologous BM-MSC	Neuroendoscope; IN + IV tandem	2	24
PAIS	Allogeneic BM-MSC	Catheter droplets	1	10
GM-IVH	Fresh breast milk; Fresh parent’s own milk	Insulin syringe drop; Oral syringe/nasal vestibule	3	48
ASD	Allogeneic MSC-exosomes; Fetal NSCs + FSC-exosomes	IN exosomes; IN + SC + inhalation + IV (mixed route)	2	2
CP	Fetal forebrain NSC	Olfactory-fissure patch	1	15
ALS	SHED-CM; UC-MSC exosomes (AlloEx)	Transnasal; Bilateral IN (Tx360®)	2	15
LBD	UC-MSC exosomes (AlloEx)	Bilateral IN (Tx360®)	1	2
KD	UC-MSC exosomes (AlloEx)	Bilateral IN (Tx360®)	1	1
CMS	UC-MSC exosomes (AlloEx)	Bilateral IN (Tx360®)	1	1
CSS	hUC-MSC exosomes	Nasal spray	1	3
HIE	Own-mother’s fresh breast milk	Intranasal syringe drops (BID)	1	10
Total	6 biologic classes	5 delivery techniques	18	153

Abbreviations: AD, Alzheimer’s disease; ALS, amyotrophic lateral sclerosis; ASD, autism spectrum disorder; CMS, congenital myasthenic syndrome; CP, cerebral palsy; CSS, Coffin-Siris syndrome; GM-IVH, germinal matrix–intraventricular haemorrhage; HIE, hypoxic-ischemic encephalopathy; KD, kennedy disease; LBD, lewy body dementia; PAIS, perinatal arterial ischaemic stroke; PD, Parkinson’s disease.

### Heterogeneity in delivery techniques

Five intranasal delivery techniques were identified across 18 patient cohorts. These included nasal spray (k = 6, n = 28; where k denotes number of studies), instillation drops or syringe delivery (k = 4, n = 64), olfactory-targeted delivery via neuroendoscope or olfactory-fissure patch (k = 3, n = 33), bilateral Tx360 applicator delivery (k = 4, n = 18), and catheter droplet delivery (k = 1, n = 10). Together, these accounted for 153 intranasally treated patients across the included studies. Delivery method, patient numbers, and cohort count per technique are presented in Table 3.

No single delivery technique was used consistently across the studies, and technique selection was strongly associated with both biologic class and disease indication throughout the cohorts. Instillation drops accounted for the largest patient exposure at n = 64. However, this was attributable almost entirely to the three preterm GM-IVH breast milk cohorts and the term HIE breast milk cohort rather than to any preference for this technique across other conditions. Nasal spray was the most frequently used technique by cohort count at k = 6, while olfactory-targeted approaches and catheter delivery were each used in only one or two cohorts. Delivery technique varied alongside biologic class, disease, and study design, the independent contribution of delivery method to any observed clinical outcomes cannot be determined from the available evidence.

### Disease-wise evidence pathways

The direction of clinical effect was assessed across all 18 patient cohorts. Fifteen reported improvements and one reported stabilization. No cohort reported worsening. Outcome measures differed substantially across diseases, interventions, and study designs. Formal pooling was not performed, and effect sizes were not compared across conditions. Two or more independent cohorts were available for AD, PD, GM-IVH, ALS, and ASD, though the same outcome measures were used across cohorts only for ALS, where both Prodromos et al. (2025) and Ueda and Seta (2022) reported the Amyotrophic Lateral Sclerosis Functional Rating Scale Revised (ALSFRS-R). For all other conditions with more than one cohort, different instruments were used, limiting direct within-condition comparisons. The remaining seven conditions were each represented by a single cohort or individual case report only.

Directional improvement was reported across neurodegenerative, neurodevelopmental, neonatal, and rare neurological disorders. These observations should not be interpreted as evidence of efficacy. Most included studies were uncontrolled, enrolled small numbers of patients, and varied substantially in biologic class, delivery method, and outcome measure. The predominance of uncontrolled designs also means that publication bias cannot be excluded, as studies reporting improvement are more likely to reach publication than those reporting no effect or worsening. All findings should therefore be treated as exploratory and hypothesis-generating rather than confirmatory.

## Discussion

There are two anatomical routes that provide support for the in the delivery of therapeutics to treat neurological diseases. From the nasal mucosa to the olfactory bulb and the larger CNS, the olfactory nerve offers a direct perivascular and axonal route (Koo et al., 2024). Along a different but similar pathway, the trigeminal nerve innervates the brain-stem nuclei and the respiratory and anterior nasal mucosa (Drath et al., 2025). According to pharmacokinetic research, drugs or any biologics can enter the cerebrospinal fluid minutes after being applied intranasally (Wu X. et al., 2025). This suggests that bulk flow through perineural spaces, rather than slowed transcellular absorption, is the primary mode of transport (Wu X. et al., 2025). In particular, the nanoscale size of exosomes and other small EVs (40–200 nm) may facilitate their uptake and transport through olfactory and trigeminal nose-to-brain pathways, including proposed axonal, perineural, and extracellular mechanisms (Gotoh et al., 2024). This is mechanistically important because intranasal administration can provide non-invasive access to the CNS while largely bypassing the BBB, which remains a major obstacle for systemic delivery of biologics to treat neurological diseases (Herman et al., 2021; Arjmand et al., 2025).

The six biologics groups discussed in this review have different mechanisms of action. This variation affects how the clinical signals should be interpreted. The most-researched type in this review, MSC-derived exosomes and EVs, transport bioactive cargo that is taken up by target cells via ligand-receptor interactions (Wu T. et al., 2025). MicroRNAs such as miR-146a, which reduces nuclear factor kappa B (NF-κB)-driven neuroinflammation, and miR-21, which inhibits pro-apoptotic signaling in brain tissue, are among this cargo (Zhang et al., 2021). Additionally, programmed death-ligand 1 (PD-L1), transforming growth factor beta (TGF-β), and interleukin 10 (IL-10) are surface immunomodulatory mediators carried by exosomes that promote macrophage polarization from the pro-inflammatory M1 to the anti-inflammatory M2 phenotype (Gu et al., 2024). Although clinical outcome data alone cannot confirm whether the same pathways were activated in the included human investigations, these characteristics provide a plausible molecular explanation for the neuroprotective signals observed in the AD and PD cohorts. The biologic cohorts of breast milk belong to a separate mechanistic group. Lactoferrin, insulin-like growth factor-1, and long-chain polyunsaturated fatty acids present in human breast milk influence neuroinflammation, synaptic plasticity, and myelination in the developing brain (Schirmbeck et al., 2022). Lactoferrin has shown antimicrobial and anti-inflammatory properties in preclinical neonatal models, and is thought to mediate part of the neuroprotective signal seen in preterm neonates following intranasal breast milk administration (Sanches et al., 2021; Schirmbeck et al., 2022; Sanches et al., 2023). Given this context, the positive findings observed in the GM-IVH cohorts appear mechanistically plausible. However, the precise proportion of bioactive constituents within breast milk responsible for the clinical signal remains undefined. This represents a critical knowledge gap, as dosage optimization and product standardization cannot be achieved without identifying the specific component(s) mediating the therapeutic effect.

Across the included studies, efficacy signals were generally favorable. Fifteen of the eighteen cohorts reported improvement, while one study, Ueda and Seta (2022), reported stabilization. In this study, the ALSFRS-R remained stable over 12 months rather than showing the progressive decline expected in ALS, so a steady score is itself a favorable signal, as no worsening was reported. These findings, however, are insufficient to establish efficacy. Most studies were uncontrolled, sample sizes were small, and outcome measures varied across cohorts, even within the same disease, precluding meaningful cross-cohort comparison. The potential for publication bias further limits interpretability. Case series and case reports were retained specifically for field-mapping and hypothesis-generation purposes given the limited body of controlled evidence for several of the 12 conditions. Taken together, these observations should be regarded as preliminary and hypothesis-generating. Definitive conclusions will require adequately powered, controlled trials with standardized outcome measures. It is necessary to explicitly describe the limits of the included studies. Interpretation is limited to feasibility and early tolerability due to several factors, including small sample sizes, the predominance of uncontrolled designs, non-standardized dose reporting, heterogeneous delivery methods, incomplete adverse-event reporting in at least two cohorts, and a high risk of publication bias. Biologic class was also confounded with disease indication, since each class was tested in a different set of conditions. No class was administered across the same conditions as another, so any apparent difference in outcome between classes cannot be separated from the difference between the diseases treated. Currently, there is a lack of uniformity in dosage units across biologic classes. Depending on the product and research, doses were expressed in milliliters, cells, particles, micrograms of protein, and percentage concentrations. Future studies should prioritize standardization. This includes standardized dosage reporting units, adverse event categorization, outcome measures selection within illness categories, and characterization of delivery techniques.

The eighteen cohorts’ compliance with the International Society for Stem Cell Research (ISSCR) 2021 Guidelines for Clinical Translation was inconsistent and frequently incomplete (Lovell-Badge et al., 2021). Twelve of the eighteen cohorts had trial registration records (Lovell-Badge et al., 2021). Only a few studies specifically validated GMP manufacturing. Stem cell–specific risks, including tumorigenicity, immunological responses, and the experimental nature of the intervention, were rarely described in sufficient detail within informed consent documents to verify adherence to ISSCR criteria. Only four registered trials incorporated formal, independent safety monitoring. Overall, clinical activity in this sector is advancing more rapidly than the infrastructure available for systematic reporting and oversight, highlighting a critical gap in governance and patient protection. Future investigations should incorporate systematic measurement of nasal deposition and, where feasible, CNS bioavailability. None of the reviewed trials provided pharmacokinetic evidence, representing a critical methodological gap. Without such data, it is not possible to establish a mechanistic link between delivery technique and clinical outcomes, nor to determine the relationship between administered dose and therapeutic impact. As all authors are employed by R3 Stem Cell LLC and/or R3 Medical Research LLC, the potential for conflict-of-interest bias should be considered alongside the methodological limitations discussed above.

## Conclusion

This systematic review compiles the available human clinical evidence on intranasal administration of regenerative biologics across 12 neurological diseases. It covers 16 articles, 153 treated individuals, and 18 patient cohorts. Across cohorts, at follow-up periods ranging from 1 to 39 months, no SAE was deemed treatment-related by investigators. Intranasal delivery of this therapeutically diverse class of biologics was reported as feasible across all five delivery methods and all six biologic types in the reviewed literature. This finding should be read as short-term tolerability in a small, highly selected population, not as evidence of safety. The near-total absence of controlled comparisons means background adverse-event rates and unreported events cannot be ruled out.

The evidence is thin and uneven. Only AD, PD, and pre-term GM-IVH have replicated within-condition data. The rest depends on single cohorts or single cases. Samples were small, most designs were uncontrolled, and the biologics, doses, delivery methods, and outcome measures differed from study to study. Cohorts could not be compared, and disease and biologic-class effects remain confounded. No study measured nasal deposition or CNS bioavailability, so the link between dose, route, and response is unknown. The finding that 15 of 18 cohorts reported improvement should not be read as a signal of efficacy. In the absence of control groups, placebo response and the natural history of the conditions studied are at least as plausible explanations as any treatment effect. Several of these conditions fluctuate or plateau on their own, and the included designs cannot separate a true treatment effect from either process.

Future progress will require standardized reporting frameworks for dosing, delivery technique, and outcome assessment. It will also require adequately powered controlled trials designed to determine whether the preliminary signals observed to date reflect a treatment effect rather than placebo response or the natural course of disease. Until such evidence exists, intranasal regenerative biologics should be considered an investigational approach with preliminary tolerability data in small, uncontrolled cohorts. Their therapeutic efficacy remains unproven, and the current evidence does not support routine clinical implementation.

## Data Availability

The original contributions presented in the study are included in the article/Supplementary Material, further inquiries can be directed to the corresponding author.
