# Supplementary material for: Intranasal delivery of regenerative biologics for neurological disorders: a systematic review of clinical studies
Source: Front Drug Deliv. 2026 Oct 16;6:1969111. doi: 10.3389/fddev.2026.1969111 (PMC13719465; doi:10.3389/fddev.2026.1969111)
Supplement: Supplementary file 1 [file Table1.docx]

**Appendix 1. Full database-specific search strategies**

Keywords only. Terms within each block were combined with OR, and the blocks were combined with AND in the database interface. Search strings are presented in plain text so that they can be copied and re-executed without truncation.

**PubMed**

***Delivery route terms:*** intranasal; transnasal; “nasal administration”; “nasal delivery”; “nose-to-brain”; “olfactory delivery”; nasal; olfactory; trigeminal; “nasal mucosa”; Nose[MeSH]; “Nasal Mucosa”[MeSH]; “Olfactory Mucosa”[MeSH]

***Regenerative biologic terms:*** “stem cell”; “stem cells”; “mesenchymal stem cell”; “mesenchymal stem cells”; “mesenchymal stromal cell”; “mesenchymal stromal cells”; MSC; MSCs; “neural stem cell”; “neural stem cells”; “extracellular vesicles”; “extracellular vesicle”; exosome; exosomes; secretome; “conditioned medium”; “conditioned media”; “breast milk”; “human milk”; “cell therapy”; “regenerative biologic”; “biological product”; “Mesenchymal Stem Cell Transplantation”[MeSH]; “Stem Cell Transplantation”[MeSH]; “Neural Stem Cells”[MeSH]; “Exosomes”[MeSH]; “Extracellular Vesicles”[MeSH]; “Regenerative Medicine”[MeSH]; “Biological Products”[MeSH]

***Neurological indication terms:*** “central nervous system”; brain; neurological; neurologic; neurodegenerative; neurodevelopmental; “Alzheimer disease”; “Alzheimer’s disease”; “Parkinson disease”; “Parkinson’s disease”; “amyotrophic lateral sclerosis”; ALS; “autism spectrum disorder”; ASD; stroke; “perinatal stroke”; “intraventricular hemorrhage”; dementia; “Nervous System Diseases”[MeSH]; “Central Nervous System Diseases”[MeSH]; “Neurodegenerative Diseases”[MeSH]; “Alzheimer Disease”[MeSH]; “Parkinson Disease”[MeSH]; “Amyotrophic Lateral Sclerosis”[MeSH]; “Autism Spectrum Disorder”[MeSH]; “Stroke”[MeSH]

***Study design and human evidence terms:*** human; patient; patients; participant; participants; clinical; trial; cohort; case series; case report; feasibility; safety; tolerability; efficacy; outcome; outcomes; follow-up; “Treatment Outcome”[MeSH]; “Clinical Trial”[Publication Type]; “Case Reports”[Publication Type]

***Limits and filters:*** Publication date 1 January 2015 to 31 March 2026; English language; human study filters applied where available. Boolean NOT animal exclusions were not used.

**Google Scholar**

***Delivery route terms:*** intranasal; transnasal; “nasal administration”; “nasal delivery”; “nose-to-brain”; “olfactory delivery”

***Regenerative biologic terms:*** “stem cell”; “mesenchymal stem cell”; “mesenchymal stromal cell”; MSC; “neural stem cell”; “extracellular vesicles”; exosome; exosomes; secretome; “conditioned medium”; “conditioned media”; “breast milk”; “human milk”; “cell therapy”; “regenerative biologic”

***Neurological indication terms:*** “central nervous system”; brain; neurodegenerative; neurodevelopmental; “Alzheimer disease”; “Alzheimer’s disease”; “Parkinson disease”; “Parkinson’s disease”; “amyotrophic lateral sclerosis”; ALS; “autism spectrum disorder”; ASD; “cerebral palsy”; stroke; “perinatal stroke”; “intraventricular hemorrhage”; dementia; “multiple sclerosis”; “traumatic brain injury”

***Human evidence terms:*** human; patient; patients; clinical; trial; cohort; case series; case report; feasibility; safety; outcome; outcomes

**ClinicalTrials.gov**

***Delivery route terms:*** intranasal; transnasal; nasal; “nose-to-brain”; olfactory

***Regenerative biologic terms:*** “stem cell”; “mesenchymal stem cell”; “mesenchymal stromal cell”; MSC; “neural stem cell”; “extracellular vesicle”; “extracellular vesicles”; exosome; exosomes; secretome; “conditioned medium”; “breast milk”; “human milk”; “cell therapy”; “regenerative biologic”

***Neurological indication terms:*** “central nervous system”; brain; neurological disorder; neurodegenerative; neurodevelopmental; Alzheimer; Parkinson; “amyotrophic lateral sclerosis”; ALS; autism; stroke; “perinatal stroke”; “intraventricular hemorrhage”; dementia

***Human evidence terms:*** clinical trial; interventional; observational; phase I; phase II; safety; tolerability; efficacy; outcome

***Registry handling:*** Registry records without an associated published report were not included as standalone evidence but were checked to identify linked or related publications.

**Chinese Clinical Trial Registry (ChiCTR)**

***Delivery route terms:*** intranasal; transnasal; nasal; “nose-to-brain”; olfactory

***Regenerative biologic terms:*** “stem cell”; “mesenchymal stem cell”; “mesenchymal stromal cell”; MSC; “neural stem cell”; “extracellular vesicle”; “extracellular vesicles”; exosome; exosomes; secretome; “conditioned medium”; “breast milk”; “human milk”; “cell therapy”; “regenerative biologic”

***Neurological indication terms:*** “central nervous system”; brain; neurological disorder; neurodegenerative; neurodevelopmental; Alzheimer; Parkinson; “amyotrophic lateral sclerosis”; ALS; autism; stroke; “perinatal stroke”; “intraventricular hemorrhage”; dementia

***Human evidence terms:*** clinical trial; phase I; phase II; safety; tolerability; efficacy; outcome; patient; participant

***Registry handling:*** Registry entries were used to identify linked human studies. Records without a linked published report were not treated as standalone evidence.
