# Supplementary material for: Intranasal delivery of regenerative biologics for neurological disorders: a systematic review of clinical studies
Source: Front Drug Deliv. 2026 Oct 16;6:1969111. doi: 10.3389/fddev.2026.1969111 (PMC13719465; doi:10.3389/fddev.2026.1969111)
Supplement: Supplementary file 2 [file Table2.docx]

**Supplementary Figure S1. Cumulative intranasal dose distribution across biologics for whole-cell studies and exosome/EV studies.** Dose-reporting heterogeneity across included studies, shown for descriptive characterization only. Panels A and B display reported dose values for whole-cell and exosome/EV studies, respectively, on logarithmic scales. Since dosing units varied across biologic classes (milliliters, cell counts, particle counts, micrograms of protein, and percentage concentrations), these panels are not standardized to a common unit and are not intended to support quantitative dose or dose-response comparison across studies or biologic classes.
